# The influence of high-temperature heating on composition and thermo-oxidative stability of the oil extracted from Arabica coffee beans

**DOI:** 10.1371/journal.pone.0200314

**Published:** 2018-07-11

**Authors:** Diana Nicoleta Raba, Dorina Rodica Chambre, Dana-Maria Copolovici, Camelia Moldovan, Lucian Octav Copolovici

**Affiliations:** 1 Department of Food Technologies, Banat’s University of Agricultural Sciences and Veterinary Medicine “King Michael I of Romania” from Timisoara, Timisoara, Romania; 2 Department of Technical and Natural Sciences, and Institute of Research, Development, Innovation in Technical and Natural Sciences, “Aurel Vlaicu” University of Arad, Arad, Romania; 3 Department of Food Control, Banat’s University of Agricultural Sciences and Veterinary Medicine “King Michael I of Romania” from Timisoara, Timisoara, Romania; University of Illinois, UNITED STATES

## Abstract

The aim of the present study was to establish the influence of high-temperature heating on the composition and thermal behavior of coffee oils obtained from Arabica green and roasted coffee beans, respectively. Morphological studies performed using scanning electron microscopy revealed the oil bodies uniformly distributed within the cells in both types of coffee beans analyzed. The obtained oils have a fatty acid composition rich in linoleic acid, palmitic acid, oleic acid, stearic acid, arachidic acid and linolenic acid. The total content of saturated fatty acids of investigated oils was 49.38 and 46.55%, the others being unsaturated fatty acids. The thermal behavior and thermo-oxidative stability of coffee oils extracted from green coffee beans and roasted coffee beans, the coffee oil high-temperature heated up to 200 °C, were investigated using simultaneous thermal analysis TG/DTG/DTA, in an oxidizing atmosphere. The data obtained for the analyzed samples depend mainly on the nature and compositions of fatty acids, and to a lesser extent on the roasting process of the coffee beans and the high-temperature heating process of the extracted oil. The chromatographic and TG/DTG/DTA data suggest that Arabica coffee oil has great potential for use in technological processes which require high-temperature heating (e.g. food industry or pastries).

## Introduction

The lipid content of the coffee is mainly determined by the coffee species: *Coffea arabica* (*Arabica*), *Coffea canephora* (*Robusta*), and *Coffea liberica*. The most popular varieties of coffee from which oil is extracted are Arabica (the average oil content is 15% on a dry basis) and Robusta (containing about 10% oil) [1–5].

The importance of coffee oil is attributed to several of its characteristics, such as its strong flavour, and its antioxidant and nutraceutical properties, which make it suitable for the food and bakery industries [6–15], and for cosmetic formulation and pharmaceutical preparations [16–25]. Also, previous studies reported that the oil extracted from spent coffee grounds represents a valuable renewable source for bio-hydrotreated fuel production [26].

The most coffee green bean lipids are found in endosperm. A small quantity is also located in the outer layer of the grain, and contains wax, oil and unsaponifiable matter, which have the role of preventing flavour volatilization during the roasting process [1, 18]. The saponifiable lipids fraction of green coffee beans contains triacylglycerols (about 75%), phospholipids and kaurane esterified diterpenes [18, 27]. The unsaponifiable fraction consists in sterols, tocopherols, free diterpenes, waxes and small amounts of other compounds with antioxidant properties, antimicrobial and antiproliferative activity [1, 28].

Data reported by Anese *et al*. [29] and Vila *et al*. [30] highlighted that the lipid fraction of coffee is stable when the beans are heated at high temperature; the heating process does not induce major changes in the fatty acids composition of oil extracted from roasted coffee beans, compared with the oil from green coffee beans. The stability of coffee oil was associated with the formation of lipid-soluble Maillard reaction products [31]. The previous GS-MS studies performed in order to investigate the fatty acids composition of coffee oil have revealed the presence of saturated (SFA) and polyunsaturated/monounsaturated fatty acids (PUFA/MUFA), from which palmitic acid (in average 46.1%) and linoleic acid (in average 32.9%) are major products. In smaller proportions, oleic acid (8.0%), stearic acid (6.6%), arachidic acid (1.9%) and linolenic acid (1.23%) were also found [14, 15, 18, 32].

Plenty of published research has shown that thermogravimetrical and differential thermal analysis methods (TG/DTG/DTA) may be applied in order to determine the thermal behavior of vegetable oils [33–41] in oxidative or inert atmospheres. These analytical techniques lead to important information regarding the thermal parameters of oils, such as initial decomposition temperature (T_onset_), thermo-oxidative stability, and mass loss of decomposition steps [33–35, 41]. In an oxidative atmosphere the vegetable oils obtained from seeds undergo, between 100–600 °C, three major degradation steps, namely: (i) decomposition of polyunsaturated acids (PUFA); (ii) decomposition of monounsaturated fatty acid (MUFA); and (iii) degradation of the saturated chains (SFA) [33, 37–39]. Additionally, the chemical composition of the oils (antioxidant compounds, lipids profile), significantly influenced the T_onset_ values [33, 35, 39, 41]. Studying the thermal behavior, in a synthetic air atmosphere, of the oil extracted from coffee beans (Robusta and Arabica varieties), Kobelnik et al. reported the recording, on the 30–650 °C temperature range, of four decomposition stages due to the oxidation processes [42].

ATR-FTIR spectroscopy analysis performed in a previous study led to the conclusion that, with regard to the oxidation stages of the extracted oil, there are no major differences between green and roasted coffee beans. The study also revealed that, after heating the oils at high temperature, there were changes of the spectral bounds in the regions 3100–3600 cm^–1^, 2800–3050 cm^–1^ and 1680–1780 cm^–1^, respectively, which were attributed to primary and secondary oxidation processes of the lipid fraction [43].

The literature currently available contains little, about the effect on the thermo-oxidative stability and fatty acid composition of the oil extracted from roasted and green coffee beans, of exposure of that oil to high temperatures. This was our main motivation for carrying out this study, which aims to assess the behavior of the lipid fraction of green and roasted Arabica coffee oils (GCO and RCO) when they are subjected to high-temperature heating (HGCO and HRCO), similar to pastry processing (at 200 °C up to 60 minutes).

In response to this lack of available information, our study addresses the following questions: (1) Does the roasting process of Arabica coffee beans, and high-temperature heating up to 200 °C of the extracted oils, induce changes in the composition of fatty acids? (2) How does the roasting process of Arabica coffee beans, and high-temperature heating up to 200 °C of the extracted oils, influence the thermal behavior and thermo-oxidative stability of the samples? (3) What is the morphological difference between the green and roasted coffee beans used for obtaining the coffee oils?

The fatty acids composition of the GCO and RCO, and of the high-temperature heated coffee oils HGCO and HRCO, was evaluated by gas chromatography-mass spectrometry (GC-MS). Also, the thermal degradation of RCO, GCO, HRCO and HGCO was investigated using thermo-analytical methods TG/DTG/DTA. The morphological features of green and roasted Arabica coffee beans used to prepare the coffee oils were determined using scanning electron microscopy.

We believe that the results obtained in this study are relevant, in respect of the possibility of using Arabica coffee oil as an ingredient in products which are processed at high-temperature, such as pastries, or in the food industry.

## Materials and methods

### Chemicals

All reagents (petroleum ether, n-hexane, toluene, methanol, n-heptane) used in the study were purchased from Sigma Aldrich (Germany) and were used without purification.

### Samples

The green and roasted coffee beans used in this study are *Coffea arabica* L. from Guaxupe Brazil, acquired from a Romanian coffee distributor (SC Global Trade SRL, 24 Constructorului St., Cornetu, Ilfov County, Romania, lot number 922/1456). The procedure for obtaining oil from green and roasted coffee beans is similar to that reported previously in [43]. Coffee beans samples were ground using a grinder (Grindomix Retsch GM 2000) and passed through a 60 mesh sieve. Soxhlet extraction was employed for 3 h to extract the oil from green (green coffee oil, GCO) and roasted coffee (roasted coffee oil, RCO) with petroleum ether (1:3, w/v). Thereafter, 20 g samples of each of the two coffee oils were heated to 200 °C for 60 minutes in atmosphere, and were then cooled at room temperature to obtain heated green coffee oil (HGCO) and heated roasted coffee oil (HRCO), respectively. All samples were kept in a refrigerator at 4 °C until analyses were performed.

### High-temperature heating treatment of coffee oil samples

20±0.5 g samples of extracted coffee oils were heated at high temperature in a forced air oven (Froilabo AC60/France, 1000 W) to 200±1 °C for 60 minutes in order to obtain HGCO and HRCO, respectively. After heating, the oils were cooled at room temperature and then they were put into dark glass bottles. All oil samples (crude and heated) were kept in a refrigerator at 4–6 °C until analyses were performed.

The treatment was performed in duplicate. Before experimental determinations the coffee oils were kept at room temperature (25 °C) for 12 h.

### Microscopic analyses of coffee beans

Crude green and roasted coffee beans were cut lengthways with a scalpel blade, localized on carbon tape placed onto support stubs, then sputter covered with gold (Automatic Sputter Coater, JEOL JFC-1300, JEOL, USA) and imaged with a LYRA 3 scanning electron microscope (LYRA 3 XMU, Tescan, Brno, Czech Republic) with secondary electron detector, at 5 kV and at different magnifications (500x, 1000x, 2000x, respectively).

### GC-MS analysis

The fatty acids contained in the sample oils (GCO, RCO, HGCO, and HRCO) were determined via their methylated derivatives obtained with the method described by Copolovici et al. in [44]. Briefly, the methyl esters of the fatty acids contained in coffee oils were obtained by treating 0.2 mL of coffee oil sample with 1.5 mL of reagent mixture (methanol/toluene/sulphuric acid: 88/10/2, v/v/v) at 80 °C for 60 minutes. Thereafter, the obtained methyl esters of the fatty acids were extracted twice, using 1 mL heptane, and were then analyzed by GC-MS. For the analysis, GC-MS equipment (Shimadzu 2010 Plus GC-MS, Shimadzu, Kyoto, Japan) with a capillary column DB 1 (30 m length; 0.25 mm i.d.; 0.25 μm film thickness) and an automatic injector was used. Helium was employed as the carrier gas, at a flow rate of 0.93 mL min^-1^. The injection temperature was 250 °C. The program of the column temperature was as follows: hold at 150 °C for 2 minutes; increase from 150 °C to 200 °C at a rate of 10 °C min^−1^; hold at 200 °C for 2 minutes; increase from 200 °C to 220 °C at 3 °C min^−1^; hold at 220 °C for 3 minutes; increase from 220 °C to 240 °C at 3 °C min^−1^; hold at 240 °C for 5 minutes. The interface temperature was 250 °C and the ion source temperature was 200 °C. The injected volume was 1 μL with a split of 1:20. The mass spectra were obtained by electron impact, using 70 eV ionization energy as the quadrupole analyzer from 31 and 400 *m/z*. The fatty acid methyl esters were identified by comparison with mass spectra, using LabSolution software (Shimadzu, Kyoto, Japan), which has as databases the NIST 14 and Wiley 09 libraries.

### Thermal analysis

The TG/DTG/DTA investigations were performed with STA 409 Luxx equipment (Netzsch, Germany), at a 30 °C—700 °C temperature range, using platinum crucibles. A dynamic air flow of 50 mL min^-1^ with 20% O_2_ was used as a decomposition atmosphere. All the experiments were conducted on samples with 25 mg mass, at β = 10 K min^-1^ heating rate. The experiments and data processing were carried out in Netzsch Proteus software.

### Stasistical analysis

The determinations of fatty acids were performed in triplicates and the results were reported as means ± standard deviation (SD). Statistical data processing was carried out by one-way analysis of variance (ANOVA), to assess the statistical significance of the observed differences. Computations of Tukey post-hoc means comparisons were included, to assess the significance of the recorded differences. The statistical analysis was carried out using the GraphPad Prism 5 software. The differences were considered statistically significant at a probability value P<0.05.

## Results and discussion

### Microscopic analyses of coffee beans

**Fig 1:**
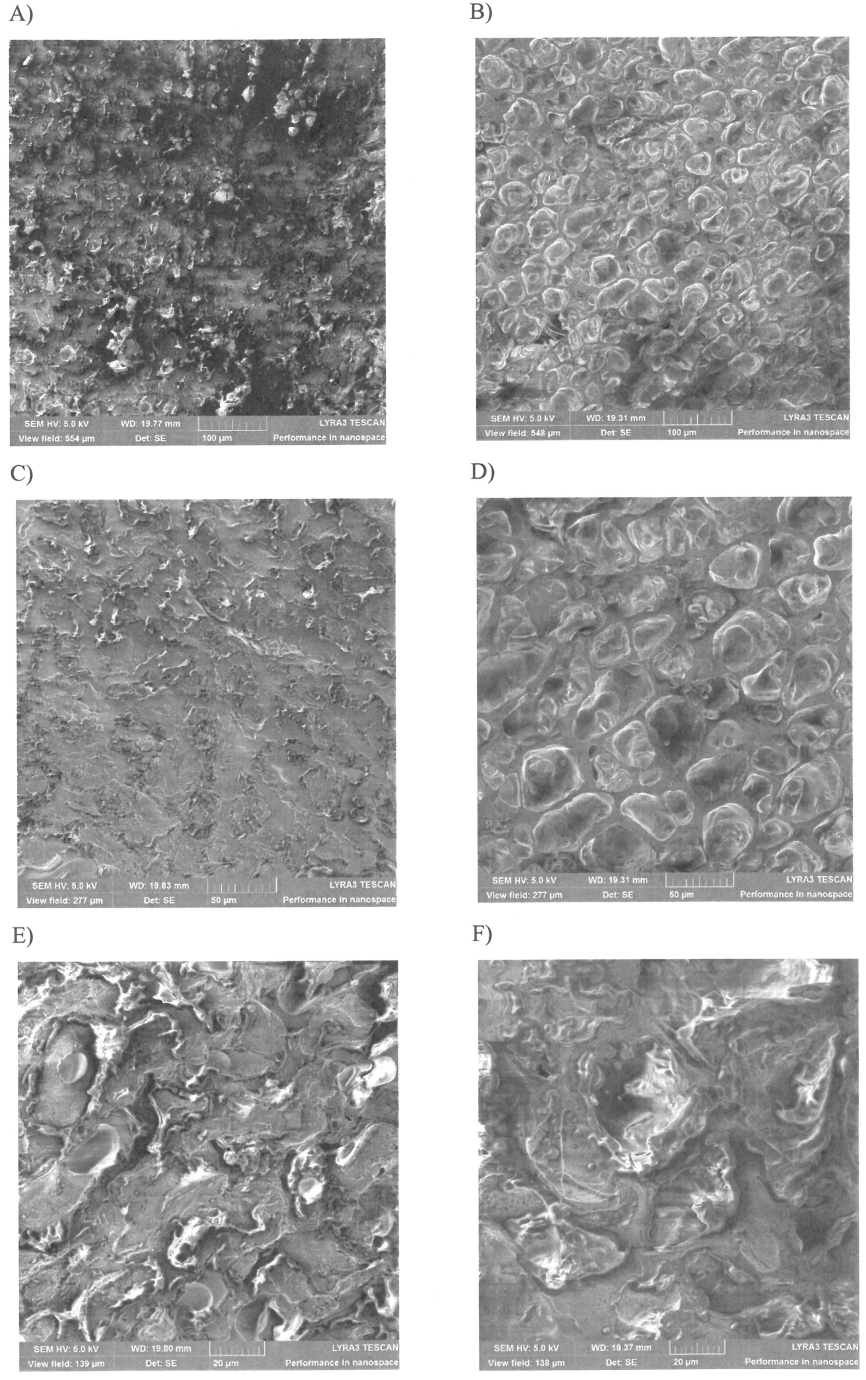
Structure of the coffee beans revealed by scanning electron microscopy: For green: (A), (C), (E) and roasted coffee beans: (B), (D), (F), at 5 kV and different magnifications: 500x for (A), (B), 1000x for (C), (D), and 2000x for (E), (F).

The microscopic observations revealed a significant difference between the green and roasted *Arabica* coffee beans. The green beans have a compact, knotty structure, and as is depicted in Fig 1 the pores are not visible, meanwhile the roasted beans are significantly less dense with a netting structure of pores. It can be seen even in the case of roasted coffee that the oils are preserved in globular vesicles, due to the integrity of the membrane.

### GS-MS analysis of coffee oil

The fatty acids that were revealed by GS-MS analysis to be contained in the investigated coffee oils are listed in Table 1. The major fatty acids of coffee oils were linoleic acid (LA, C18:2) and palmitic acid (PA, C16:0), followed by oleic acid (OA, C18:1), and stearic acid (SA, C18:0). The percentages of arachidic acid (AA, C20:0) and linolenic acid (LA, C18:3) are between 0.92–3.25%. In any case, it has been demonstrated that the free fatty acids content of freshly harvested green coffees is very low [45]. The total content of SFA in coffee oils obtained from green and roasted beans is 49.38% and 46.55% respectively. Such values are in line with other determinations which have been reported for coffee oils obtained from Arabica [14, 45, 46] and Robusta [47, 48] coffee beans. The composition of palmitic, arachidic, oleic, linoleic and linolenic acids is not significantly affected by the roasting treatment. The same trend has been shown for almost all *Arabica* coffee using Canonical varieties analysis [45]. In contrast, the percentage of stearic acid significantly decreased due to the roasting treatment.

**Table 1:** Fatty acids composition (%) of the investigated coffee oils (green coffee oil, GCO; roasted coffee oil, RCO; heated green coffee oil, HGCO; heated roasted coffee oil, HRCO).

Fatty acid / (% ± SD)	GCO	RCO	HGCO	HRCO
Palmitic acid (PA) C16:0 (SFA)	38.3 ± 1.9^a^	37.4 ± 1.3^a^	38.7 ± 2.4^a^	36.6 ± 1.2^a^
Stearic acid (SA) C18:0 (SFA)	7.89 ± 0.34^a^	6.70 ± 0.22^b^	9.01 ± 0.42^c^	8.32 ± 0.31^ac^
Arachidic acid (AA) C20:0 (SFA)	3.24 ± 0.27^a^	2.41 ± 0.17^b^	3.96 ± 0.11^c^	3.25 ± 0.18^a^
Oleic acid (OA) C18:1 (MUFA)	9.34 ± 0.50^ab^	8.91 ± 0.39^b^	9.23 ± 0.41^ab^	10.03 ± 0.36^a^
Linoleic acid (LA) C18:2 (PUFA) n-6	39.9 ± 4.8^a^	43.6 ± 3.7^a^	37.9 ± 2.5^a^	40.4 ± 3.1^a^
Linolenic acid (LNA) C18:3 (PUFA) n-3	1.25 ± 0.37^a^	0.92 ± 0.19^a^	1.19 ± 0.11^a^	1.33 ± 0.31^a^
% Total SFA	49.38	46.55	51.68	48.17
% Total MUFA+PUFA	50.58	53.42	48.29	51.76

Means were compared by Tukey’s multiple comparison *post-hoc* test (*n* = 4). Different letters indicate means that are statistically different at *P <* 0.05.

The high-temperature heating treatment of oils led to an increase in total percentage of SFA for both samples: from 49.28% for GCO to 51.68% for HGCO, and from 46.55% for RCO to 48.17% for HRCO, while the total percentage of PUFA was decreased: from 50.58% for GCO to 58.29% for HGCO, and from 53.42% for RCO to 51.76% for HRCO (Table 1). The heating of oils at 200 °C did not significantly change the percentage composition of palmitic acid, linoleic acid, oleic acid and linolenic acid, nor that of oleic acid, in case of heating of GCO. Meanwhile, a significant increase in the composition of stearic acid and arachidic acid was determined. The heat treatment of fats induces modifications of fatty acids with two or three double bonds (which have low thermal stability): these may undergo isomerization from *cis* to *trans* form. Such formation of *trans* fatty acids has been observed during thermal treatment of cocoa beans [49]. Heating treatment caused, in flaxseed oil, a significant increase of the relative content of palmitic, stearic and oleic acid and a decrease in the relative content of linolenic acid. Moreover, at 105 °C, changes occur in the relative content of different fatty acids, but in a very small amount [50]. The same results have been obtained for sunflower oil, cottonseed oil and palm oil during frying of French fries at 180 °C [51]. In this case, total PUFAs tended to decrease while SFA increased, which has been explained by interaction between food products and bath oil. In our case, the unsaturated fatty acids decrease, maybe due to their oxidation in the roasting process.

### TG/DTG/DTA analysis

**Fig 2:**
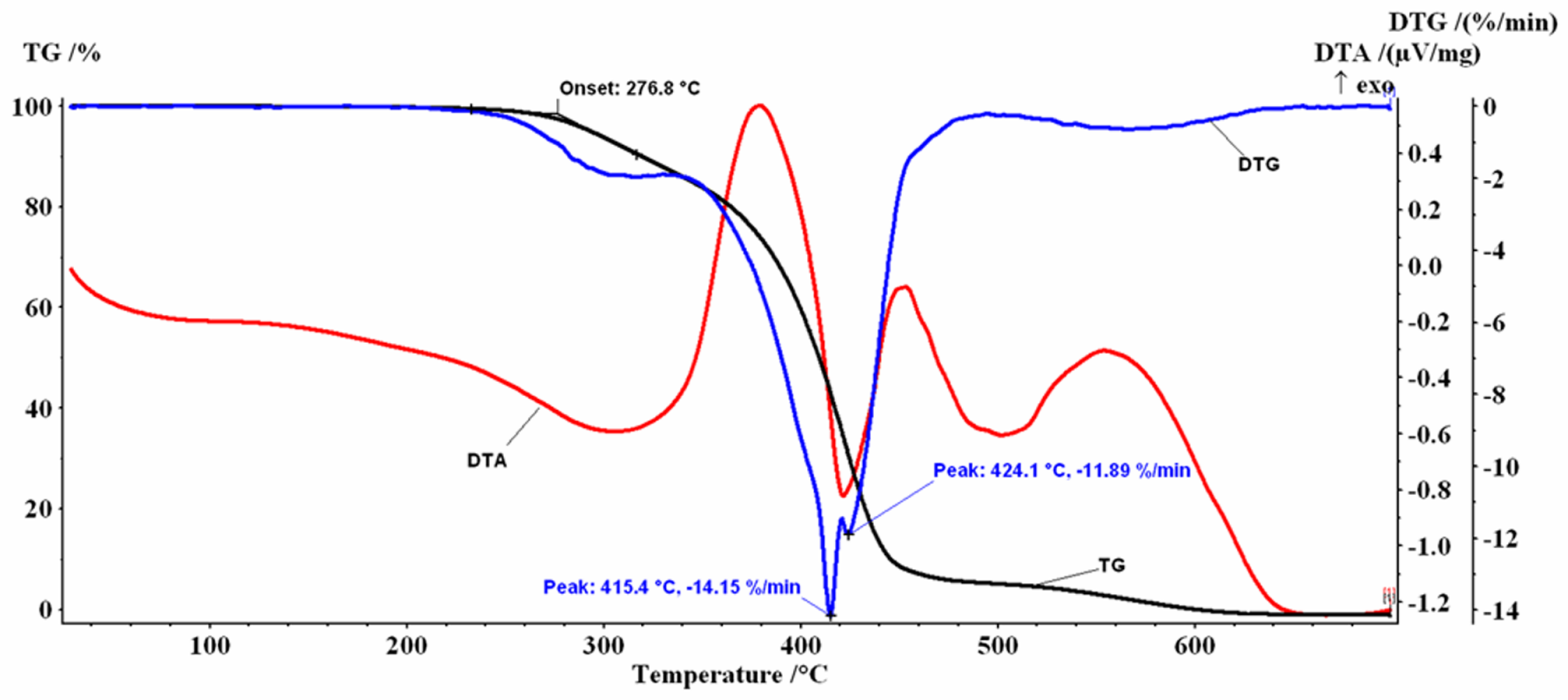
TG and DTG/DTA profiles for GCO.

**Fig 3:**
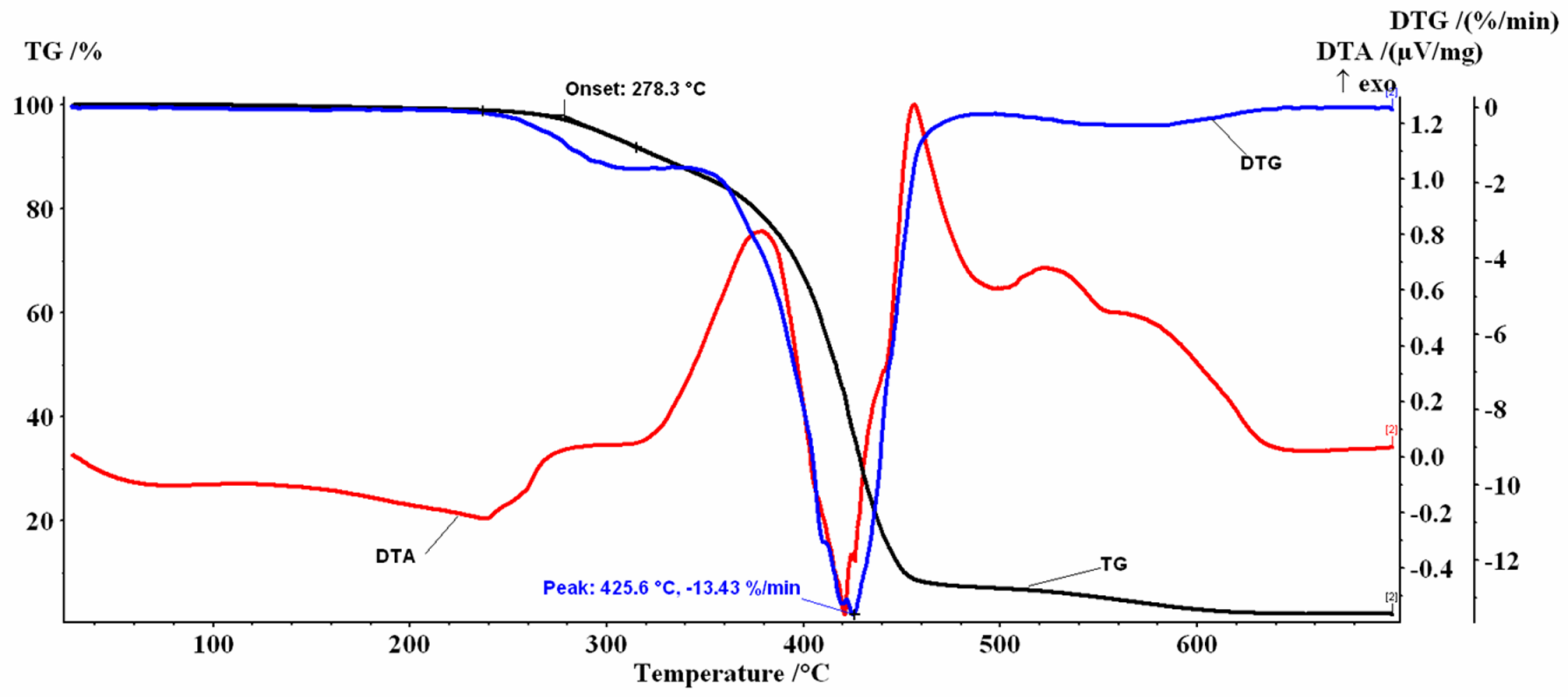
TG and DTG/DTA profiles for RCO.

**Fig 4:**
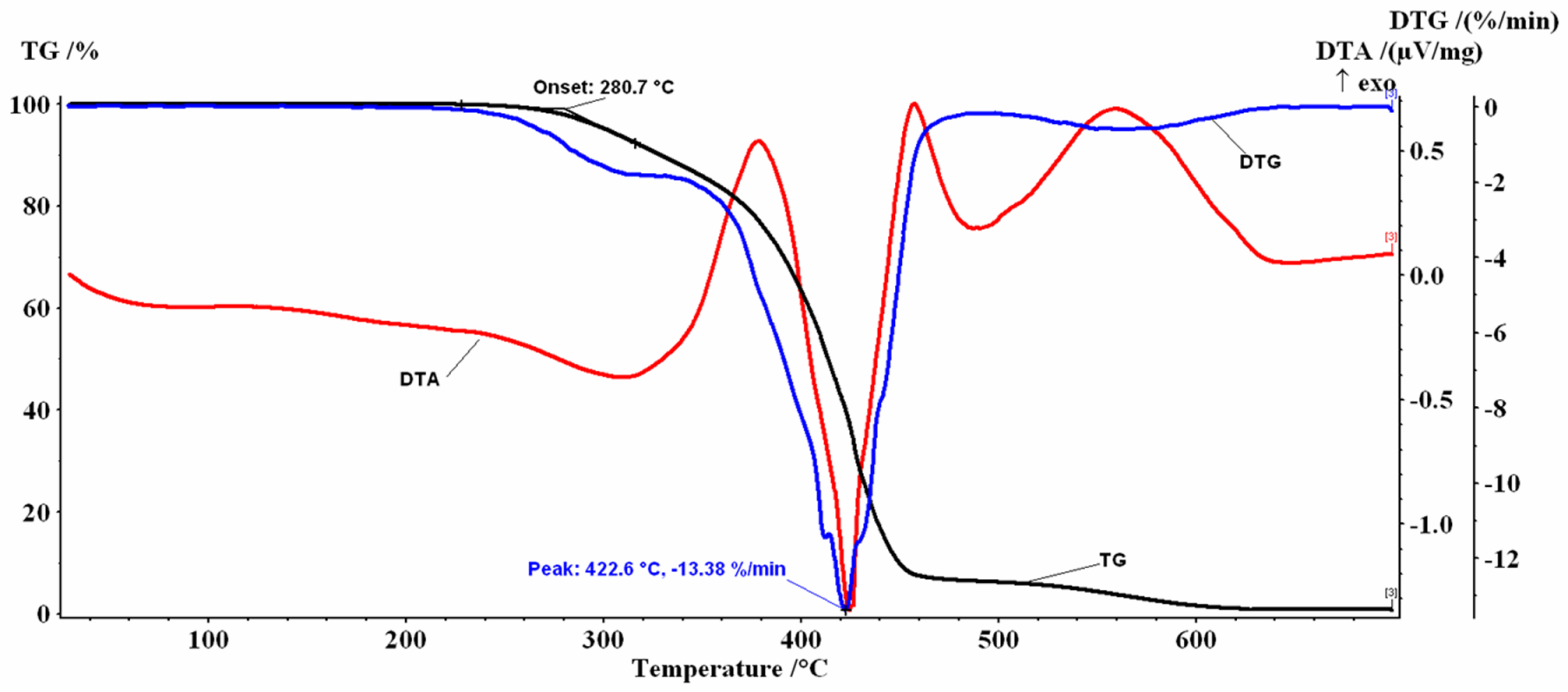
TG+DTG/DTA curves for HGCO.

**Fig 5:**
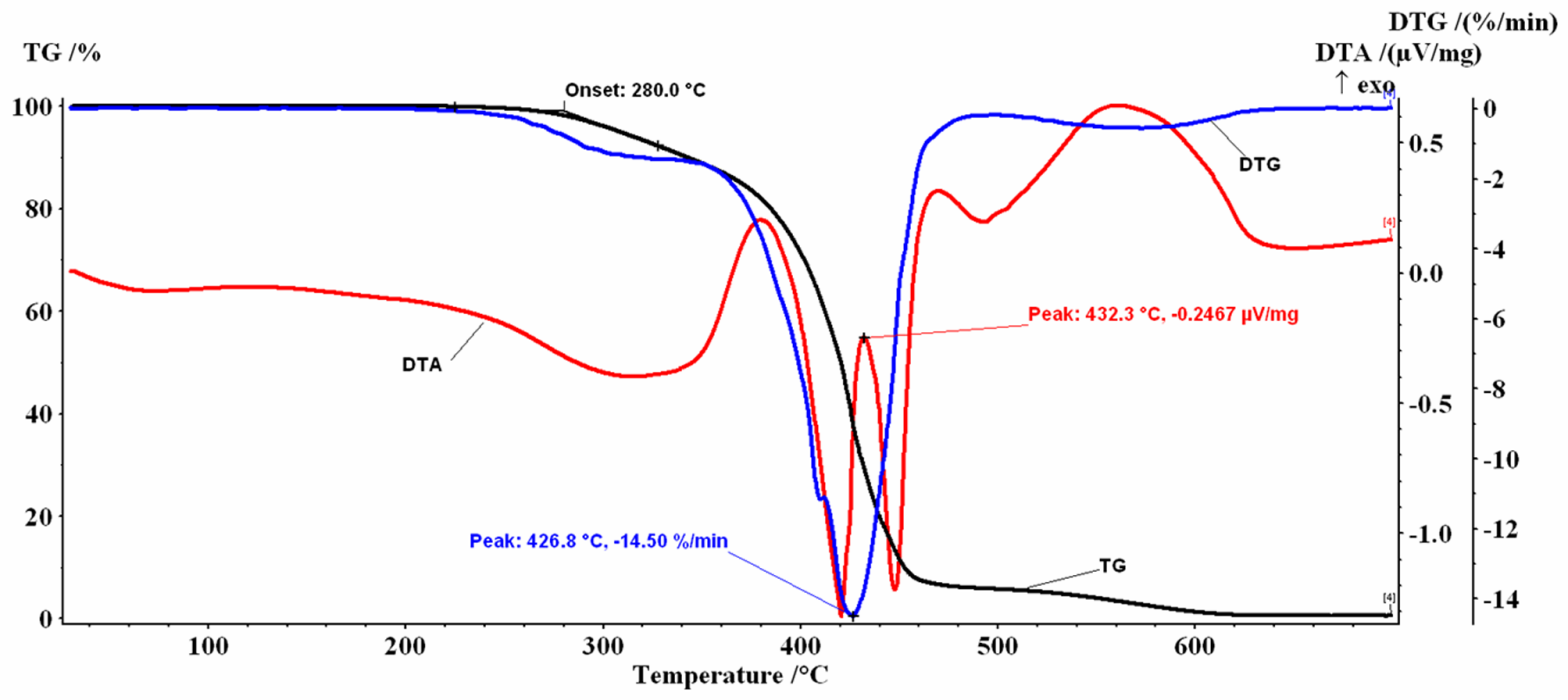
TG+DTG/DTA curves for HRCO.

Similar profiles for the thermogravimetric TG/DTG/DTA curves were obtained for the coffee oils investigated (Figs 2–5).

In the 30–700 °C temperature range the analyzed samples, in air flow, presented several decomposition stages accompanied by mass-loss (%Δm), behavior similar to that reported in the literature both for the oil extracted from green coffee beans (GCO and HGCO) and for the oil obtained from roasted beans (RCO and HRCO) [22].

The mass-loss stages recorded are mainly due to the thermal degradation of the saturated (SFA) and unsaturated fatty (PUFA+MUFA) acids which constitute more than 99% of oil mass, as was previously described in detail [52].

At the beginning of the recorded TG curves a *plateau* is observed that attests to the absence of solvent in the samples and to their thermal-oxidative stability up to approx. 276 °C. The first decomposition stage was recorded between 220 °C and 365 °C, the second decomposition stage was recorded between 340 °C and 525 °C and the last one between 500 °C and 630 °C.

In dynamic oxidative atmosphere, for all samples, a small residual mass was noticed, probably caused by the process of obtaining the oils. The thermal decomposition data resulting from TG/DTG curves are listed in Table 2.

**Table 2:** TG/DTG decomposition data of the coffee oils samples.

Sample	1^st^ step		2^nd^ step		3^rd^ step		Residual mass (%)
	Temperature range (°C)	Δm_1_ (%)	Temperature range (°C)	Δm_2_ (%)	Temperature range (°C)	Δm_3_ (%)	
GCO	220–340	8,89	340–500	85,62	500–618	5,2	0,29
RCO	240–360	11,24	360–520	81,78	520–625	5,99	0,99
HGCO	225–350	7,99	350–505	85,92	505–620	5,29	0,8
HRCO	245–365	10,79	365–525	83,23	525–630	5,33	0,65

Based on the correspondence between the mass-loss values shown in Table 2 and the data from Table 1, the 1^st^ step can be attributed to the decomposition of unsaturated acids (PUFA+MUFA) which have the double bond, mostly LNA C18: 3, but also a fraction of LA C18:2 and OA C18: 1. The RCO sample that had the highest PUFA+MUFA content (53.42%) also showed the highest value of mass-loss at this stage (Δm_1_ = 11.24%). Mass-loss values decreased with decreased content of unsaturated fatty acids, up to 7.99% for the HGCO sample.

Mass-loss values recorded for unheated oil samples (GCO and RCO) were slightly higher than for high-temperature heated samples (HGCO and HRCO). A more significant increase of mass-loss values (~ 3%) can be seen in the case of oil samples extracted from roasted coffee beans than in the case of the green coffee beans. These results could suggest that a series of processes associated with the formation of lipid-soluble Maillard reaction products occurred, during the roasting of the beans or the high-temperature heating of the extracted oil, which induced a small change in the composition and thermal behavior of the samples. The RCO is a brown liquid characterized by an intense and pleasant aroma. The color of the oil is mainly due to the presence of Maillard reaction products that are obtained during the seed roasting process [23].

For the 2^nd^ step, the form of the recorded TG/DTG/DTA curves is a complex one. For example, for the GCO sample the DTG curve shows two maxima located at T_DTG_ = 415.4 °C, respectively at T_DTG_ = 424.1 °C. On the DTA curve two intense exothermic peaks were recorded (Fig 2). A similar behavior was recorded for the RCO, with T_DTG_ = 425.6 °C, (Fig 3) and for the HGCO, with T_DTG_ = 422.6 °C (Fig 4).

Regarding the HRCO sample with T_DTG_ = 426.8 °C it can be seen that, on the 2^nd^ step temperature range, the DTA curve shows three intense exothermic peaks (Fig 5). This behavior suggests that, in the 340 °C– 525 °C range, several overlapping oxidative decomposition processes take place, which are difficult to separate.

These processes are due both to the decomposition of the PUFA fraction (mostly LA C18:2) and the MUFA fraction (OA C18: 1) remaining from the 1^st^ step, with which the SFA decomposition overlapped, and to the thermo-oxidative combustion of the degradation products formed. The mass-loss values increase from Δm_2_ = 81.78% for the RCO sample that had the lowest SFA content (46.55%), to Δm_2_ = 85.92% for the HRCO oil sample. The values recorded for oil samples extracted from green coffee beans (GCO and HGCO) were higher than those recorded for roasted coffee beans (RCO and HRCO).

In the 3^rd^ step, total oxidation of the carbonaceous residues formed on the 2^nd^ step occurs. The mass-loss values and the characteristic temperatures (T_DTG_ = 560 °C) of the 3^rd^ decomposition step are comparable for all analyzed oils. An exothermic effect on the DTA curves was recorded.

The 1^st^ decomposition step of the oil samples is the most important one in the thermal-oxidative stability study. According to the values of the initial decomposition temperature (T_onset_) afferent to this stage, the investigated samples showed a stability to oxidation up to approx. 276.8 °C -278.3 °C (Figs 2 and 3), respectively up to 280.0 °C -280.7 °C (Figs 4 and 5). The 2–3 degree difference observed may be due to the small differences in the fatty acid composition of the oils induced by the Maillard reaction that occurred during bean roasting or during high-temperature heating of the oils up to 200 °C, and also to the small differences found in the recorded mass- loss values.

The oil extracted from Arabica green or roasted coffee beans has a thermo-oxidative stability up to about 270 °C. The heat treatment of the beans during the roasting process did not significantly affect the composition in fatty acids. Therefore, this lipid system could be a valuable natural ingredient for the food products industry, where technological steps without thermal treatments or with thermal treatments below 250 °C are required.

## Conclusions

In the present study we showed that the morphology of roasted coffee beans is preserved in comparison with that of green coffee beans, and that the oil bodies are uniformly distributed within the cells. The oil obtained from green and roasted coffee beans has a fatty acid composition rich in linoleic acid and palmitic acid, followed by oleic acid, stearic acid, arachidic acid and linolenic acid. The total content of saturated fatty acids (SFA) in coffee oils obtained from green and roasted beans is 49.38 and 46.55%, respectively, the others being unsaturated fatty acids. The thermal behavior and thermo-oxidative stability of coffee oil extracted from the Arabica green and roasted beans (GCO and RCO), respectively of the coffee oil high-temperature heated up to 200 °C (HGCO and HRCO), were investigated using simultaneous thermal analysis TG/DTG/DTA, in oxidizing atmosphere. In the 30–700 °C temperature range the analyzed oil samples, in air flow, presented several overlapped decomposition stages accompanied by mass-loss. The thermal behavior and thermo-oxidative stability of the investigated samples depended mainly on the nature and compositions of PUFA, MUFA and SFA and less on the roasting of the coffee beans or high-temperature heating of the extracted oil. The TG/DTG/DTA data suggest that the Arabica coffee oil has great potential to be used in technological processes which require high-temperature heating, such as those employed by the food industry, or in the production of pastries.

## Abbreviations

DTA: differential thermal analysis curves
DTG: differential thermogravimetric analysis
GC-MS: gas chromatograph coupled with mass spectrometer detector
GCO: green coffee oil
HGCO: heated green coffee oil
HRCO: heated roasted coffee oil
MUFA: monounsaturated acids
One-way ANOVA: One-way analysis of variance
PUFA: polyunsaturated acids
R: correlation coefficient
RCO: roasted coffee oil
SD: standard deviation;
p: probability
SEM: scanning electron microscopy
SFA: saturated fatty acids
TG: thermogravimetric analysis
